# Primary care functional features and their health impact on patients enrolled in the Shanghai family doctor service: a mixed-methods study

**DOI:** 10.7189/jogh.15.04007

**Published:** 2025-01-10

**Authors:** Yang Wang, Hua Jin, Hui Yang, Yang Zhao, Yi Qian, Dehua Yu, Hai Fang

**Affiliations:** 1 Department of General Practice, Research Center for General Practice, Yangpu Hospital, School of Medicine, Tongji University, Shanghai, China; 2 Shanghai General Practice and Community Health Development Research Center, Shanghai, China; 3 School of Public Health, Peking University, Beijing, China; 4 China Center for Health Development Studies, Peking University, Beijing, China; 5 Department of General Practice, Monash University, Victoria, Australia; 6 The George Institute for Global Health, University of New South Wales, Sydney, Australia; 7 The George Institute for Global Health, Beijing, China; 8 School of Public Health, Hangzhou Normal University, Hangzhou, China

## Abstract

**Background:**

While research in multiple countries confirms that primary care functional features significantly improve patient health, China's primary care system differs markedly due to unique structural and contextual factors. This study aims to measure and explore the functional features experienced by patients received family doctor contract service in the past year, evaluating the impacts and pathways of these primary care features on health outcomes.

**Methods:**

We employed a mixed-methods explanatory sequential design. In the quantitative phase, we randomly selected 2118 residents from 12 primary care institutions. The intensity of functional features was assessed using the Person-Centered Primary Care Measure (PCPCM), and their association with levels of EuroQol Visual Analogue Scale (EQ VAS) was evaluated through multilevel modelling. In the qualitative phase, a qualitative description approach was used, conducting 24 focus groups with a total of 85 patients to gather in-depth information about their experiences with functional features and perceived health impacts. Finally, the quantitative and qualitative data were integrated using meta-synthesis and joint display methods to validate, interpret, and expand the results.

**Results:**

The average PCPCM score was 3.65, with subdomain scores ranging from 3.39 to 3.83. Qualitative findings confirmed the quantitative results regarding the intensity and manifestation of features like accessibility, coordination, and relationship-building. However, discrepancies were noted in features such as comprehensiveness, integration, and family and community context. Additionally, two new functional features, ‘being appreciated’ and ‘being cared for,’ were identified. The quantitative results also showed that higher PCPCM scores were positively associated with EQ VAS levels (odds ratio (OR) = 1.18; 95% confidence interval (CI) = 1.03–1.35, *P* < 0.001). Furthermore, qualitative results revealed six key pathways supporting the beneficial effects of local primary care functional features on health maintenance and improvement.

**Conclusions:**

This study demonstrates high functional scores for Shanghai's family doctor services and highlights a positive association between primary care functionality and population health. These features and their health benefits are deeply shaped by the local social and health care context. This confirms the progress of Shanghai's primary care development and underscores the need for further exploration of primary care functional features across China, along with the development of tools tailored to local conditions to better measure and improve primary care quality and health outcomes.

**Keywords:**

primary healthcare; primary care; quality measurement; population health; mixed method research; China

Achieving specific functions in primary care is often effective in improving population-level health outcomes and enhancing other aspects of primary care performance [1–7]. While the domains – referred to as ‘features’ [5], ‘characteristics’ [3], ‘attributes’ [1], or similar terms – may vary across theories [3,8,9], certain common areas like the ‘4Cs’ (first contact access, comprehensiveness, continuity, and coordination) are widely considered essential functional features and broadly applicable to primary care systems worldwide [5].

Since the 1980s, as China transitioned from a planned economy to a market economy, its primary care system has gradually been influenced by both the Chinese government’s distinct, direct administrative control and patients' freedom to choose providers in an open health care market [10,11]. On one hand, the government’s direct administrative regulation ensures that services provided by primary care institutions are distributed more equitably compared to those offered by large general hospitals, while requiring the provision of a set amount of preventive medicine and public health services to nearby community residents [10,12]. Since 2011, primary care delivery has gradually shifted from a passive model, where primary care institutions waited for patients to seek care, toward the government-promoted ‘family doctor contract service’ model [13]. This model encourages family doctor teams in primary care institutions to provide continuous primary care services to approximately 1000–2000 residents from nearby communities or villages, with whom they sign annual service agreements, fostering ongoing relationships between general practitioners and patients [14,15]. The scope of care focuses primarily on the prevention, intervention, and treatment of common and chronic diseases, particularly for older adults and those with chronic conditions [12–14]. According to plans set by the National Health Commission, the coverage of family doctor contract services in China is expected to exceed 75% of the total population by 2035 [16].

On the other hand, from the perspective of health care recipients and their choices, the Chinese medical market does not implement a gatekeeping mechanism for primary care. Residents are free to select their health care providers from any primary care institutions and general hospitals nationwide, including specialist departments within large tertiary hospitals [17]. On a national scale, compared to large hospitals, China's primary care institutions notably lack advanced technology, equipment, and pharmaceutical resources, creating a distinct divergence from the primary care infrastructure found in Europe and North America [10,18]. Additionally, the scope of clinical services offered by primary care practitioners is limited, focusing primarily on basic and low-tech care [19,20]. As a result, this seemingly ‘fair’ open-market competition has led to a long-standing erosion of the patient population served by primary care institutions, which has also negatively affected the family doctor services provided by general practitioners working within these institutions [20,21]. While many residents sign free health care service agreements with nearby family doctor teams as required by the government, they often bypass primary care and seek treatment directly from specialists at large general hospitals, especially less healthy individuals or those of higher socioeconomic status [21–23].

The unique and evolving primary care systems in China highlight the need to investigate how functional features manifest and influence health outcomes in the region, including the potential emergence of new, valuable features, to support the measurement and improvement of primary care quality both locally and in similar regions. Currently, some studies have used translations and validated versions of the Primary Care Assessment Tool (PCAT) to assess the levels of primary care functional features among patients in different regions of China [24–31]. However, as a tool originally developed for the primary care context and services in the USA, a key limitation of using the PCAT in quantitative surveys of local primary care patients is its limited ability to capture primary care features beyond the predefined domains [32]. Moreover, the majority of these studies use the levels of functional features measured by PCAT as the main outcome, with only one study conducted in Tibet demonstrating a positive correlation between the strength of primary care functional features and self-rated health status on a five-point Likert scale [26]. This indicates that not only is our understanding of potential localised functional features within China’s primary care system limited, but we also lack more reliable evidence regarding the relationship between these features and health outcomes for the patients they serve.

In this study, we aim to assess the levels of primary care functional features perceived by patients enrolled in the family doctor contract service in Shanghai and explore how these features are experienced by local patients. Additionally, we seek to examine whether higher levels of perceived functional features are associated with better health outcomes among contracted patients, as well as the pathways through which these features might influence those outcomes. We chose to begin our work in Shanghai due to its economic advancement, with a per capita gross domestic product (GDP) of 26 700 USD in 2022 [33], making it more comparable to high-income countries than other regions of China. Furthermore, since 2010, Shanghai has been at the forefront of developing its primary care system and implementing the family doctor contract service programme [34]. The practical efforts of the local government and health care professionals – such as the implementation of the family doctor contract service and the continuous enhancement of primary care clinical capacities and scopes [35,36] – strengthen our confidence that Shanghai is one of the regions in China where residents are most likely to experience the health benefits provided by well-functioning primary care features.

## METHODS

**Figure 1:**
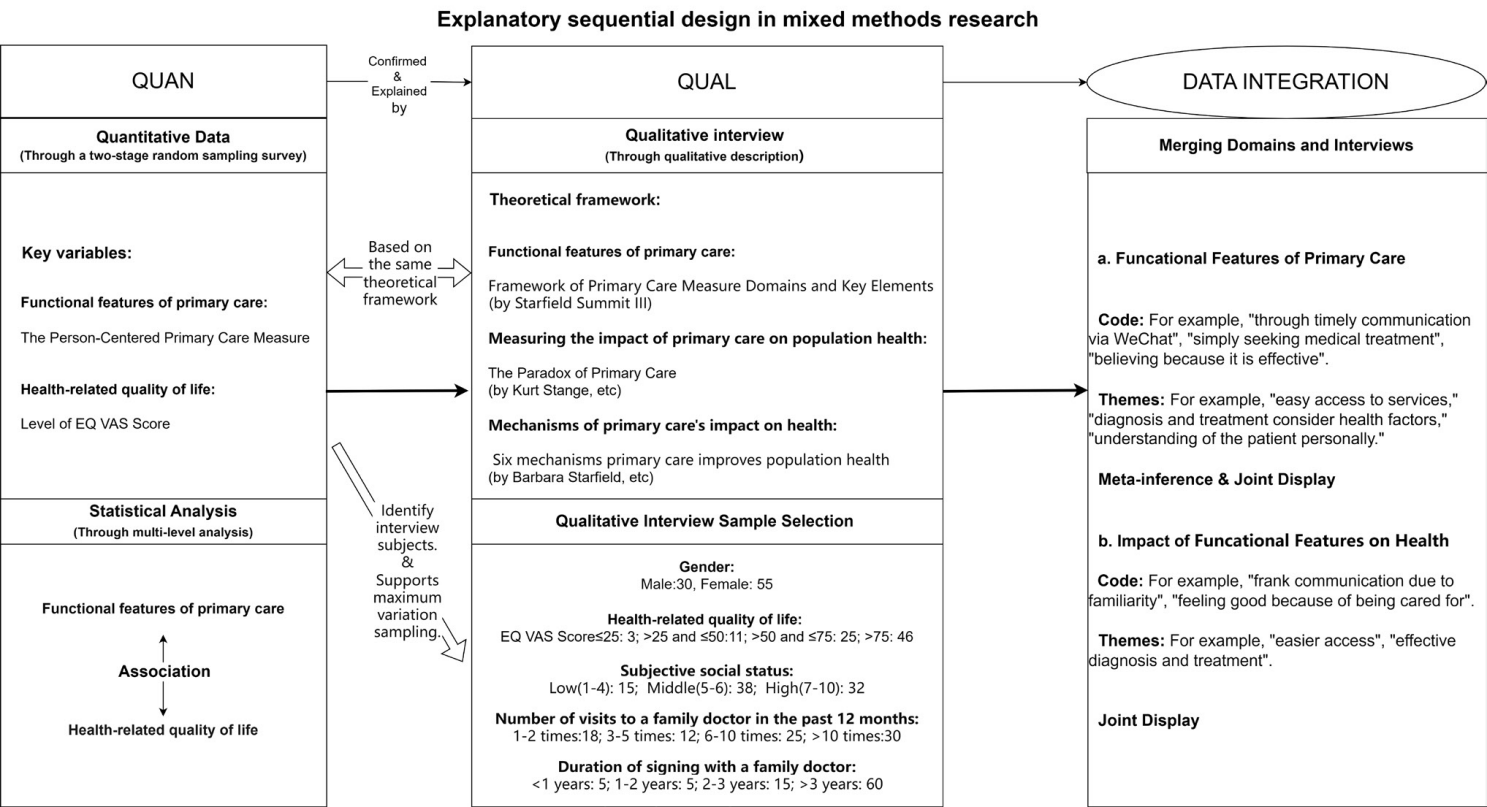
The methodological detail of mixed-methods explanatory sequential design. QUAL – qualitative study section, QUAN – quantitative study section.

Addressing the research questions, we implemented a mixed-methods explanatory sequential design [37] (**Figure 1**). By synthesising both quantitative and qualitative results, we aimed to validate and interpret the quantitative data through the qualitative data, thereby deriving more reliable and insightful findings.

### Quantitative research section

Our target population consisted of patients enrolled in family doctor services in Shanghai, accounting for approximately 30–40% of the city’s permanent resident population [38]. Adhering to the Person-Centered Primary Care Measure (PCPCM) guidelines for selecting survey participants [39], our inclusion criteria were:

1) Shanghai residents enrolled in family doctor contract services

2) individuals aged 18 and older

3) those able to understand the study's ethical guidelines and provide consent, either in writing or verbally.

We excluded individuals with severe illnesses or mental disorders, as they might be unable to independently and clearly understand or respond to the survey content. Additionally, we made the difficult decision to exclude residents enrolled in the family doctor service who had not utilised it in the past year, primarily to ensure the accuracy of our measurements. Although these individuals are technically part of the enrolled population, their lack of recent service use means their PCPCM responses would not reflect actual, recent experiences. However, given that studies in China show primary care utilization is closely linked to care quality and patient experience [21,22], excluding this group might result in slightly inflated reported functional features compared to those of the entire enrolled population.

We established a two-stage random sampling methodology [40] (Figure S1 in the **Online Supplementary Document**). According to 2022 data, Shanghai had 248 primary care institutions providing primary care and family doctor contract services, with each centre serving around 32 000 individuals [41]. In a preliminary survey focused on EuroQol Visual Analogue Scale (EQ VAS) involving 100 patients, we formed four groups of 25, achieving an average EQ VAS score of 79.33 with a standard deviation of 22.87. The mean EQ VAS score for individual groups was 1983.25, with an intergroup variance of 351.19. Setting a 95% confidence interval and a sampling precision of 0.1, and utilising the calculation formula


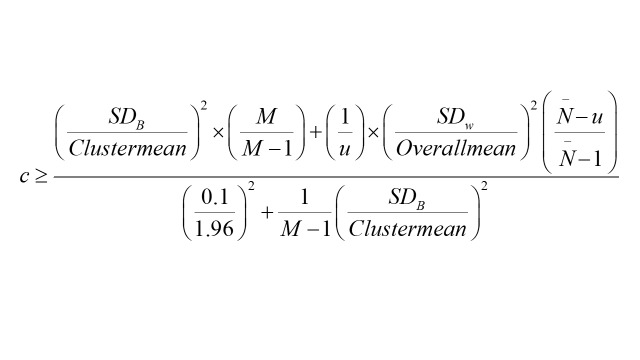
,

it was determined that surveying at least 12 primary care institutions in Shanghai was necessary, aiming for 150 subjects per center and expecting an 85% valid response rate, to achieve a minimum total sample size of 2118 patients.

In our study, we categorised Shanghai’s primary care institutions into the upper and lower 50% tiers according to their overall clinical quality scores, as reported by the Shanghai General Practice Clinical Quality Control Center [42]. We further segmented these groups based on geographical location into urban, suburban, and rural categories. Utilising computer-generated random numbers, we then randomly selected two facilities from each subgroup to ensure a diverse range of settings. Within each chosen facility, a designated institutional coordinator was tasked with randomly selecting 250 residents who met the inclusion and exclusion criteria from the list of residents registered with family doctors, thereby fulfilling our sample size requirements. If a selected resident refused to participate in the study, they were excluded from the survey participants.

Building on previous research, we developed a survey questionnaire and conducted a preliminary study with 60 patients at a suburban primary care facility in Shanghai in July 2023, after which we refined the questionnaire. The core variables included the intensity of the functional features of primary care, assessed through the PCPCM [43], which has been adapted and validated locally for Chinese patients with family doctor contracts (Table S1 in the **Online Supplementary Document**). We chose this scale because its eleven items succinctly capture the essential beneficial aspects of primary care, reflecting the direct perceptions and feedback of primary care service users regarding its 11 widely recognised functional features. This makes it more flexible and adaptable than methods that gather responses from specific populations to fixed scenarios within a particular country's primary care context and then judge the intensity of the functional features of primary care. The outcome variables were measured using the EQ VAS, a component of the Chinese version of the EQ-5D-5L [44], one of the most classic and widely used tools for assessing patient-reported outcomes. This outcome was chosen because the EQ VAS score more directly reflects respondents' perceptions of their current health status compared to the EQ-5D utility index [45]. We selected 14 covariates primarily based on two previous local studies that examined the causal relationship between primary care functional features and health status [26,46]. Additionally, we consulted relevant literature on the development and initial testing of the PCPCM and PCAT [32,43]. Detailed information about these covariates is provided in Table S2 in the **Online Supplementary Document**.

In October–November 2023, under the coordination of an institutional coordinator, trained surveyors conducted face-to-face surveys using electronic questionnaires developed by Questionnaire Star. Completed surveys were automatically uploaded to a server for analysis. Subsequently, authorised by the primary care institutions, surveyors accessed and anonymised patient medical records from the past three months for supplementary questionnaires, integrating these with the main survey data.

For statistical analysis, we began with descriptive statistics to depict the demographic characteristics of the participants. We computed the mean and standard deviation across the 11 domains of the PCPCM. Subsequently, multilevel modelling was employed, designating primary care institutions as the first level and patients as the second, to evaluate the correlation between PCPCM scores and EQ VAS scores. The selection of covariates was guided by the identification of Minimal Sufficient Adjustment Sets (MSAS) from causal directed acyclic graphs (DAGs), aiding in the estimation of both total and direct effects [47] (Figure S2 in the **Online Supplementary Document**). Our analytical process adopted a sequential two-model strategy, initially focusing on a model encompassing solely the main outcome. The subsequent model, aimed at assessing total and direct effects, included seven covariates as ancestors to both exposure and outcome: gender, age, household registration, education, income, subjective social status (SSS), and chronic diseases, along with two exposure-specific variables: number of visits and contract duration.

In the sensitivity analysis, considering that a patient's health condition might inversely affect their perceived strength of primary care functional features – such as patients with severe conditions potentially rating the comprehensiveness of family doctor services lower – we employed two variables theoretically closely related to PCPCM scores and unlikely directly associated with EQ VAS levels as instrumental variables. These variables include the availability of family doctors to provide services during the weekend and the provision of telephone health consultations to patients after hours. Following the assessment of endogeneity, weak instrumental variables, and overidentification tests, we scrutinised the causal relationship using the two-stage least squares (2SLS) instrumental variable approach [48]. In Table S3 in the **Online Supplementary Document**, we report more details regarding the rationale for selecting these two variables and the results of the instrumental variable validity tests conducted on them.

Additionally, we used a similar multilevel model to analyse four key subgroups: residents with low SSS (scores 1–4), moderate SSS (scores 5–6), high SSS (scores 7–10), and residents with chronic diseases. Statistical analyses were carried out with Stata 17.0 SE (StataCorp LL, College Station, TX, USA, 2021), setting the significance threshold at *P* ≤ 0.05.

### Qualitative research section

We adopted a qualitative descriptive approach [49,50] and selected 24 focus groups [51], consisting of 85 patients in total, for interviews. The background characteristics of the participants are shown in Table S4 in the **Online Supplementary Document**. These patients had agreed to participate in qualitative interviews subsequent to their involvement in the quantitative survey, as depicted in Figure S1 in the **Online Supplementary Document**. The theoretical framework we used, formulated during the 2018 Starfield Summit III, focused on the domains and essential elements of primary care measurement [3]. This theory, grounded in the scholarly contributions of Starfield and Stange [1,2], asserts that the intrinsic value of primary care is primarily attributable to its unique functional features. These features, in synergy, enhance its overall effectiveness, especially concerning health outcomes.

After the quantitative phase, we randomly selected a primary care facility for an initial series of interviews with five patient groups, each characterised by varied demographic traits. Following these preliminary interviews, we utilised maximum variation sampling to choose 15 patient groups from five additional facilities, considering critical aspects like gender, health status, SSS, the number of visits to a family doctor in the past year, and the total duration of enrolment with a family doctor.

One week before the interviews, we engaged institutional coordinators to secure a small round-table meeting room. A researcher employed at a primary care research institution and a general practitioner with part-time research responsibilities, both proficient in qualitative study and with expertise in primary care functional features, facilitated focus group sessions lasting between 50 to 80 minutes. Participants were initially briefed about the study, completed consent forms, agreed to audio recordings, and were assured of the confidentiality of their responses and their freedom to withdraw at any time. Open-ended questions, derived from the original concepts of the 11 domains of the PCPCM and three closely related theoretical frameworks [3,43] and adapted to local primary care scenarios, were outlined, with thematic areas detailed in Table S5 in the **Online Supplementary Document**. As we conducted additional focus groups, questions were continually adjusted – added, removed, or modified – based on the latest interview results to achieve data saturation across specific sub-themes and facilitate the emergence of fresh insights. After each thematic discussion, an interviewer summarised the received information, presenting it to elicit immediate feedback from all participants, thus ensuring reflexivity and reducing biases linked to the researchers' personal experience and interpretations.

During the interviews, the facilitator consistently encouraged each group member to express their views and ensured that everyone had roughly equal and ample time to share their opinions. Primary care personnel from the involved facilities were not present during the interviews to enhance open and unrestricted dialogue between the patients and the interviewers. After conducting 20 focus group interviews, we found that no new codes, sub-themes, or themes emerged. We then conducted an additional four focus group interviews, consulting participants on sub-themes with relatively less data, more sensitive codes, and questions related to new themes beyond the theoretical frameworks. After confirming that no new information was obtained in these final interviews, we concluded that data saturation had been reached, as Sandelowski refers to as ‘informational redundancy’ [52].

Recordings were transcribed and manually cleaned on the same night of the interview, with continuous, iterative content analysis conducted using MAXQDA 2020 (VERBI GmbH), following strategies to enhance rigor in qualitative description [53]. Two experienced researchers analysed the focus group interview texts, with findings cross-checked to ensure study reliability. Analysis involved immersive reading and reflection on the transcripts to form a comprehensive understanding of the research themes. Line-by-line analysis was used to identify and code meaningful text units related to the study's main topics, with notes documenting insights and critical reflections. Since the topics of the qualitative interviews were partially shaped by the theoretical framework used in the qualitative section, specifically the domains of the PCPCM, the data analysis conducted by two researchers followed a continuous, parallel process of deductive and inductive coding. They systematically extracted a series of codes from the qualitative transcripts and reflective notes, categorising them under various sub-themes that emerged both within and beyond the original framework. As sub-themes accumulated, new themes could potentially emerge from those outside the initial framework.

Throughout the interview process, after every 1–2 interviews, the two researchers compared and discussed the codes and sub-themes they had generated, including new themes, to ensure consistency in the focus of subsequent interviews. Once all interviews were completed, the researchers held a final group discussion to resolve any remaining discrepancies in the codes and sub-themes. An independent researcher with extensive experience in primary care was then invited to review and validate the descriptive summaries and representative quotations used to present the final themes.

### Integration

The theoretical frameworks for the survey and interviews were synchronised, facilitating the integration of quantitative and qualitative data. Following the meta-synthesis steps outlined by Creswell [54] and employing Gutman's joint display method [55], we structured the results from both studies into two distinct sections. The first section combined the intensity and manifestation of primary care functional features, leading to the creation of meta-inferences that deepened our understanding of the overall state of local primary care functionalities. Building on this foundation, the second section was dedicated to exploring the health impacts and underlying pathways.

### Ethical statement

This study strictly adhered to the Declaration of Helsinki. All residents participating in the questionnaire survey were informed about the ethical aspects of the study by the surveyors and provided verbal informed consent. Additionally, all residents participating in the focus groups signed written informed consent forms. Ethical approval for this study was obtained from the ethics review board at Peking University, with the approval number: IRB00001052-23077.

## RESULTS

The response rate of the quantitative survey was 73.67%. The respondents' sociodemographic characteristics and health status are detailed in (**Table 1**). The mean EQ VAS score was 72.17 (standard deviation (SD) = 14.52). Of these respondents, 28.6% agreed to participate in subsequent qualitative interviews. Table S4 in the **Online Supplementary Document** shows the characteristics and health status of the participants included.

**Table 1:** Sociodemographic characteristics and health of the patients included

Variables	No.	%
Gender		
*Male*	916	41.45
*Female*	1294	58.55
Age		
*<35 y*	71	3.21
*≥35 y and <55 y*	214	9.68
*≥55 y and <65 y*	335	15.16
*≥65 y and <75 y*	946	42.81
*≥75 y*	644	29.14
Hukou (household registration)		
*Rural*	321	14.52
*Urban*	1889	85.48
Education		
*Did not finish primary school*	103	4.66
*Finished primary school*	401	18.14
*Finished middle school*	631	28.55
*Finished high school*	660	29.86
*Graduated from college*	415	18.78
Income		
*<40 000 yuan*	626	28.33
*≥40 000 yuan and <60 000 yuan*	713	32.26
*≥60 000 yuan and <80 000 yuan*	451	20.41
*>80 000 yuan*	420	19.00
Subjective social status		
*Score 1–4*	266	12.04
*Score 5–6*	1085	49.10
*Score 7–10*	859	38.87
Suffered chronic diseases		
*Without chronic diseases*	216	9.77
*With chronic diseases*	1994	90.23
Physical activity		
*Does not meet WHO recommended levels*	1292	58.46
*Meets WHO recommended levels*	918	41.54
Alcohol consumption		
*Do not drink alcohol*	1970	89.14
*Drink less than one drink per day*	177	8.01
*Drink one or more drinks per day*	63	2.85
Smoking status		
*No smoking*	1970	89.14
*Low dependence*	189	8.55
*Moderate or high dependence*	51	2.31
Obesity status		
*Not overweight*	1331	60.23
*Overweight or obese*	879	39.77
Health insurance		
*No medical insurance*	7	0.32
*Moderate insurance scheme*	902	40.81
*High-coverage insurance scheme*	1301	58.87
Duration of contract with the family doctor		
*Within one year*	167	8.26
*1–2 y*	171	8.46
*2–3 y*	259	12.82
*Over three years*	1424	70.46
Number of visits to the family doctor in the past 12 mo		
*1–2 times*	329	14.89
*3–5 times*	552	24.98
*6–10 times*	659	29.82
*More than 10 times*	670	30.32
EQ VAS score		
*≤25*	20	0.90
*>25 and ≤50*	170	7.69
*>50 and ≤75*	1067	48.28
*>75*	953	43.12
Total	2210	100.00

EQ VAS – EuroQol Visual Analogue Scale, WHO – World Health Organization

**Table 2** employs a joint display to showcase the quantitative results, qualitative insights, and meta-inferences. Quantitatively, the average score for the PCPCM was 3.65 (range: 1–4), indicating generally high ratings across its 11 domains. Domain 1 received the highest score at 3.83, while Domain 6 registered the lowest at 3.39. Qualitatively, patients often contrasted their experiences at primary care institutions with those at large tertiary hospitals. The existence and intensity of certain primary care functions, such as accessibility, coordination, relationship building, advocacy, and disease and illness management and prevention, were confirmed. These findings were also enriched by qualitative data into how local family doctor contract services actualise these functions. For example, the widespread use of instant online consultation software was noted for enhancing accessibility, while strategic ‘reverse coordination’ was employed to integrate specialists into primary care settings, improving comprehensiveness and coordination. Additionally, the extensive delivery of health education and regular lifestyle modification reminders for community residents highlighted the community orientation aspect.

**Table 2:** Perceived primary care functional features by residents enrolled in family doctor contract services in Shanghai

Functional features	Quantitative results	Qualitative results		Meta-inference
**PCPCM domain**	**Mean (SD)**			
		**Residents experience**	**Quotation**	
1. My practice makes it easy for me to get care	3.83 (0.38)	Family doctors are more accessible than large hospitals, offering easier appointment scheduling and prescription services. Patients can conveniently contact them via phone or WeChat for quick consultations and timely responses. In some areas, family doctors visit communities and even provide home health care. While the cost difference is minor, seeing a family doctor is generally less expensive than visiting a large hospital.	Interviewee 4D: Actually, family doctors are very convenient. Why? As we age – I signed up with a family doctor about five or six years ago – our health naturally starts to decline, and issues become more frequent, making regular hospital visits necessary. Signing up with a family doctor just makes life easier. There are several reasons. If I feel unwell on weekends, I can consult with my family doctor via WeChat, because I don't have anywhere else to turn, not being medically trained myself. I think having a family doctor is very good, very good indeed. They do as they say, visiting the community every Tuesday. If you need medication, you just say what you need in the WeChat group, and they'll deliver it promptly, which is fantastic.	Confirmed: Patients who have signed up with family doctors can easily access primary care services from them. Expanded: This ‘ease of access’ is facilitated by closer geographic proximity, easier appointment scheduling, the convenience of phone and online software consultations, more frequent community and home visits by family doctors, and slightly lower medical and medication prices than those at large tertiary hospitals.
2. My practice is able to provide most of my care	3.78 (0.45)	Family doctors offer a broad range of basic health services, including initial consultations, diagnosis of common illnesses, prescription of medications, ongoing management of chronic conditions, basic physical exams, and health education. However, they often face challenges with serious illnesses, trauma, emergencies, specialized diseases, severe chronic disease complications, and treatments requiring advanced equipment. Additionally, there can be issues with physician capabilities, medication supplies, and equipment availability.	Interviewee 4B: It depends on the severity of the illness. For example, if you suddenly pass out, I wouldn't go to a family doctor because they don't have the equipment. If you need a CT scan or an MRI, in my understanding, you have to go to a specialized hospital. Like, if we're talking about injuries, broken bones, or brain injuries, I'd think of Hospital X for broken bones and Hospital Y for brain injuries. Family doctors are good in my book, but I'm cautious about their medical facilities or level of medical care because I rarely get sick. I figure they're a bit below the doctors at top-tier hospitals. Interviewee 6A: With chronic diseases, like basic conditions such as hypertension or diabetes, you're better off going to a family doctor if it's well-managed. But, for instance, in my family, someone has kidney issues. If the kidney proteins or creatinine levels go up, that's beyond what a family doctor can handle. You'd need to see a specialist in a large tertiary hospital for that. They can address the issue. Once everything is stable again, you can go back to your family doctor for ongoing management. But if your condition isn't stable, you definitely need a hospital doctor to get it under control first.	Discordant: Despite quantitative results showing that the vast majority of patients believe family doctors can provide most of the health services they need, qualitative results reveal clear limitations in the scope of services family doctors can offer. Expanded: The free medical market's filtering of patient preferences impacts how patients perceive family doctors' services: many patients, sceptical of the capabilities of family doctors, opt to directly seek care at large tertiary hospitals instead. Thus, only those who believe their health issues can be adequately addressed seek care from family doctors.
3. In caring for me, my doctor considers all the factors that affect my health	3.68 (0.54)	Family doctors are well-acquainted with the long-term health of familiar patients and often discuss lifestyle factors. However, they have limited knowledge of infrequent visitors. While some tailor advice and prescriptions to a patient's health history, it is more common to prescribe medication and treat illnesses directly.	Interviewee 18D: Like with me and my medication, my family doctor knows my situation well. He would advise against taking unnecessary medications and help improve my condition. My mom used to have lung issues, and our family doctor would suggest how she could exercise and make a lot of reasonable recommendations based on our conditions. Interviewee 1A: For regular matters like eating and sleeping, family doctors might know more about their frequent patients. For someone like me, who doesn't visit often, they might not have as much insight. When you do go for a visit, they'll ask about your situation to get a clear understanding, mainly because they're familiar with patients who visit them often.	Discordant: Qualitative results indicate that family doctors do not universally consider factors affecting patients' health in their services as much as quantitative results might suggest. Expanded: The natural selection of patient preferences also may impact the assessment of this feature. Family doctors may be more aware of and consider health-related factors for patients who frequently seek care, but this is challenging to achieve for patients who do not visit regularly.
4. My practice coordinates the care I get from multiple places	3.62 (0.57)	Electronic health records have significantly helped family doctors become familiar with their patients' treatment information from other facilities. They can refer patients to partnered tertiary hospitals, and some medications typically available only at larger hospitals can be prescribed by family doctors and mailed to patients, reducing the need for cross-institutional referrals. Additionally, some tertiary hospital doctors are required to provide outpatient services at primary care institutions, further integrating health care services. However, many patients are unaware of these referral options and believe it’s no more convenient than going directly to a tertiary hospital.	Interviewee 2C: The family doctor can see where you've been prescribed medication and treated. Everyone has a file in the computer, and you can also tell him yourself. The day I went to the hospital to see him, he could look it all up on his computer. Interviewee 6D: If I have an issue that can't be addressed here, the doctor will tell you and suggest where else you can go. For example, after signing up with them, they might mention there's a second-tier or third-tier hospital available. They'll usually direct you first to the second-tier hospital, and if that doesn't work, you can decide on your own to go to the third-tier hospital or elsewhere. If you bypass their suggestion and feel that the doctor here isn't good or you have doubts, sometimes I go directly to other large tertiary hospitals – it's up to you. Interviewee 1C: Just to add, now in large tertiary hospitals, like X, Y, and Z, the doctors and specialists come to our community health centre regularly. If you need to see a specialist, you can tell the family doctor, and they'll help you schedule an appointment. You can see them right in the community health centre. So, there's even less need to go to the large tertiary hospital.	Confirmed: Qualitative results support quantitative findings that family doctors can access information about patients' visits to other institutions, aiding in referrals to relevant partner hospitals and obtaining medications. Expanded: Beyond traditional coordination, Shanghai's primary care system features a ‘reverse coordination’ mechanism, where instead of transferring patients to large tertiary hospitals, specialist doctors are moved to primary care institutions.
5. My doctor or practice knows me as a person	3.63 (0.55)	Family doctors are well-acquainted with patients who frequently visit them, especially those with serious conditions. They generally understand these patients' medical histories and medication use. Some family doctors also know their patients' behavioral habits and family backgrounds, often becoming seen as friends and companions.	Interviewee 1B: Once there's mutual understanding, and he knows your medical conditions, it's like having a good friend. Now, we're just like good friends with our family doctor and his team. If there's a problem, I just ask them, and they always respond. Interviewee 4C: My family doctor is familiar with my high blood pressure issues. But it's impossible for him to know every tiny detail because they're busy, and there are so many elderly in our community – he can't cover everything. He likely pays more attention to people who are sicker and visit more frequently. Still, I have great respect for their service and care for us.	Confirmed: The qualitative results support the quantitative findings that there is a clear personal relationship between family doctors and patients. Expanded: This feature's quantitative measurement is also influenced by the patients' preferences and frequency in seeking care from family doctors. Family doctors are more familiar with patients who visit more often, whereas those who seldom seek care may not consistently maintain a contract with a family doctor.
6. My doctor and I have been through a lot together	3.39 (0.75)	Many family doctors have long-term contractual relationships with their patients. This doctor-patient relationship is maintained through frequent visits to the family doctor and the proactive, ongoing communication from the doctor via phone calls. Special experiences that occur during this process make them more familiar with each other.	Interviewee 18B: There's definitely been help over the (nine years of the contract). Signing up with a family doctor has been an overall process, a continuity. The family doctor is quite familiar with my condition and provides timely solutions to any issues. Interviewee 2C: When I call, it's just me and the family doctor talking. He asks me, “How have you been recently? Good, what medication are you taking, what do you take for high blood pressure, any issues with your gallbladder, are you taking your gallbladder tablets? We eat well every night, bowel movements are good,” he basically calls once a month, it's all pretty good.	Confirmed: The qualitative finding corroborates the quantitative findings, indicating that family doctors and patients often have a long-term relationship.
7. My doctor or practice stands up for me	3.65 (0.58)	Many residents believe their family doctors protect their interests, which motivates them to engage in long-term care. This trust is built on the genuine efforts of family doctors to provide effective medical services and their familiarity with patients. It also contrasts with the impersonal attitudes often encountered in large tertiary hospitals.	Interviewee 21D: The doctors treat us patients like we're 'one of their own'. We get that feeling, and we see the doctor as 'one of us' too. When there's something we don't understand, we go to the doctor. Of course, there are limits and tiers to their expertise; we can't expect family doctors to have the highest level of technical skill, after all. Yet, our doctors treat you like family, very warmly. Interviewee 15D: It's not just that the large tertiary hospitals are crowded, as the lady before me said. What you consider a big problem is a small one to them. ‘This minor issue, we see a lot of it here, several times a day,’ they think. They consider it a minor surgery, which is trivial to them.	Confirmed: The qualitative results support the quantitative data, showing that most patients trust their family doctors' efforts to protect their health and well-being. Expanded: From the patients' perspective, the indifferent attitude of specialist physicians in large tertiary hospitals towards their health concerns contrasts with the relatively warm attitude of family doctors. This contrast may lead patients to believe that family doctors are more dedicated to protecting their health interests.
8. The care I get takes into account knowledge of my family	3.61 (0.60)	Many family doctors lack an understanding of their patients' family backgrounds and do not incorporate this information into their diagnostic processes. Those who do consider family information often have specific characteristics. For example, the entire family might have a contract with the same doctor, the patient may frequently seek treatment and be well-acquainted with the doctor, or the doctor might be a resident of the same community or village.	Interviewee 14C: The family doctor does ask questions. Like today, when I came in, I happened to meet with the family doctor, and he asked why I was alone. I told him my husband went to a gathering with friends. You can tell from these little things because when we come to get medication, we usually come together, my husband and I. Sometimes he asks why someone didn't come or if they're feeling unwell. He's quite caring about these details. Interviewee 5A: We wouldn't discuss things like how many people are in the household or how many children there are with the family doctor. Our main concern is getting treated or getting medication. We don't talk about family backgrounds.	Discordant: The qualitative findings significantly differ from the quantitative results, pointing out that family doctors do not commonly incorporate considerations of family background factors into their practices as the quantitative data suggests.
9. The care I get in this practice is informed by knowledge of community	3.58 (0.62)	Family doctors regularly provide health education to community residents. Some even take on leadership roles in public health affairs and carry out public health initiatives. However, unless a family doctor has a specific contract with a community and operates a nearby clinic, or is a local resident, their understanding of community health factors is limited and rarely integrated into their clinical practice.	Interviewee 1B: My current family doctor has become the vice-chair of our neighborhood's health and welfare Committee. The chair is the director of the street committee, and there are several other members. The committee mobilizes residents to participate in sanitation efforts, cleaning up pollution or other issues in certain areas. Together, we all keep the public health aspects of our neighborhood clean. Interviewee 18A: He doesn’t understand our community's situation because I am a ‘fan signer’ (meaning residents choose to sign up with a family doctor they like, rather than the one responsible for their community). This doctor is not the one assigned to our neighborhood. He knows me personally, but he doesn’t know much about my community or family.	Discordant: The qualitative findings diverge significantly from the quantitative data, highlighting that family doctors do not uniformly integrate considerations of community background factors into their practice as the quantitative results might suggest. Expanded: One pathway through which family doctors engage in community health work involves conducting collective health education for community residents and organizing group public health activities within the community.
10. Over time, my practice helps me to stay healthy	3.70 (0.51)	Family doctors ensure the continuous provision of medical care, medication, and behavioral interventions for their patients, which may help maintain their health. However, some patients believe that the fundamental power to improve health lies within themselves, and what family doctors provide is merely external support.	Interviewee 4B: Family doctors can help you get your hypertension medication in a timely manner. Even though I take it sporadically, they continue to refill my prescription and occasionally call to check on how well my blood pressure is controlled, which is enough for me. What more could you want? It’s unrealistic to expect them to become full-time private doctors. We need to take initiative for our own care, and what they are able to offer now is already very good.	Confirmed: Family doctors maintain the health of many patients by consistently providing resources and primary care services.
11. Over time, my practice helps me to meet my goals	3.67 (0.51)	Family doctors do not help patients create personalized health plans but rather focus on implementing the national protocols for chronic disease management and control. Their approach tends to be more ‘top-down’ rather than ‘bottom-up’.	Interviewee 5A: My family doctor calls me regularly. ‘How's your heart been lately, how's your blood pressure, are you taking your medication? Don't forget to take your meds,’ but there’s no help in creating any health plan for me.	Discordant: Qualitative research reveals that family doctors are not pursuing patient-initiated ‘my goals’ but rather government-mandated ‘our goals.’ Local residents' understanding of issues related to PCPCM may not clearly distinguish between ‘I want to implement a health plan’ and ‘the government wants me to implement a health plan.’
S1. This feature is not included in the PCPCM.	No quantitative data	Many family doctors provide services that make patients feel valued and respected. Their patience, positive attitude, and meticulous care sharply contrast with the hurried, indifferent, and sometimes arrogant approach of some doctors in large tertiary hospitals. However, not all family doctors are patient and friendly towards their patients.	Interviewee 1A: Our family doctors listen to you slowly, explain what medication you should take, and if you have any reactions to the medication, they'll even change it for you. large tertiary hospitals don't usually do that; they're not as meticulous. If you haven't explained your condition well, they'll prescribe medication right away. Dr X is really great. Interviewee 5C: To be fair, because large tertiary hospitals are just too busy. They have to see hundreds of people a day, and before you've finished talking, they've hurried you along and sent you out. Is it my fault for not speaking clearly? Large tertiary hospitals are just too big; they see people from all over the country.	Expanded: We define this dimension as ‘Be Appreciated,’ which means that, compared to some large tertiary hospitals, the services provided by family doctors make patients feel valued, respected, and supported with meticulous patience by their physicians.
S2. This feature is not included in the PCPCM.	No quantitative data	Patients feel societal care from family doctors' services, which stem from their friendly attitude and proactive, continuous contact. This is especially significant for the elderly.	Interviewee 15A: It improves your mood to think, ‘Why is my family doctor so nice, even taking the time to care about me, right?’ It feels like now the family doctor is so kind, and then they contact me, and I ask about the medication I'm taking. It really lifts my spirits. Sometimes, I think being sick isn't always about the illness; mood is very important.	Expanded: We describe this dimension as ‘Being cared for,’ indicating that patients feel socially cared for and loved through the friendly and emotional service provided by their family doctor team.
Total	3.65 (0.50)	All the content listed above.		

PCPCM – person-centred primary care measure, SD – standard deviation

Furthermore, qualitative observations revealed potential response biases, stemming from how respondents' choices influenced their perceptions of certain functional feature domains, such as comprehensiveness, integration, family and community context, and goal-oriented care. Those who are more familiar with and in greater need of family doctor services are more likely to seek care from them, making them more likely to be included in the sample, which often results in higher evaluations of related functional features. Additionally, subtle differences in how respondents actually interpret theoretical concepts like ‘provide most of my care,’ ‘the care I receive takes into account knowledge of my family/community,’ and ‘meet goals’ may have contributed to an overestimation of these domains in the PCPCM. Lastly, the qualitative research uncovered two new themes highly valued by local patients but absent from the original 11 domains of the PCPCM: ‘being appreciated’ and ‘being cared for’.

**Figure 2:**
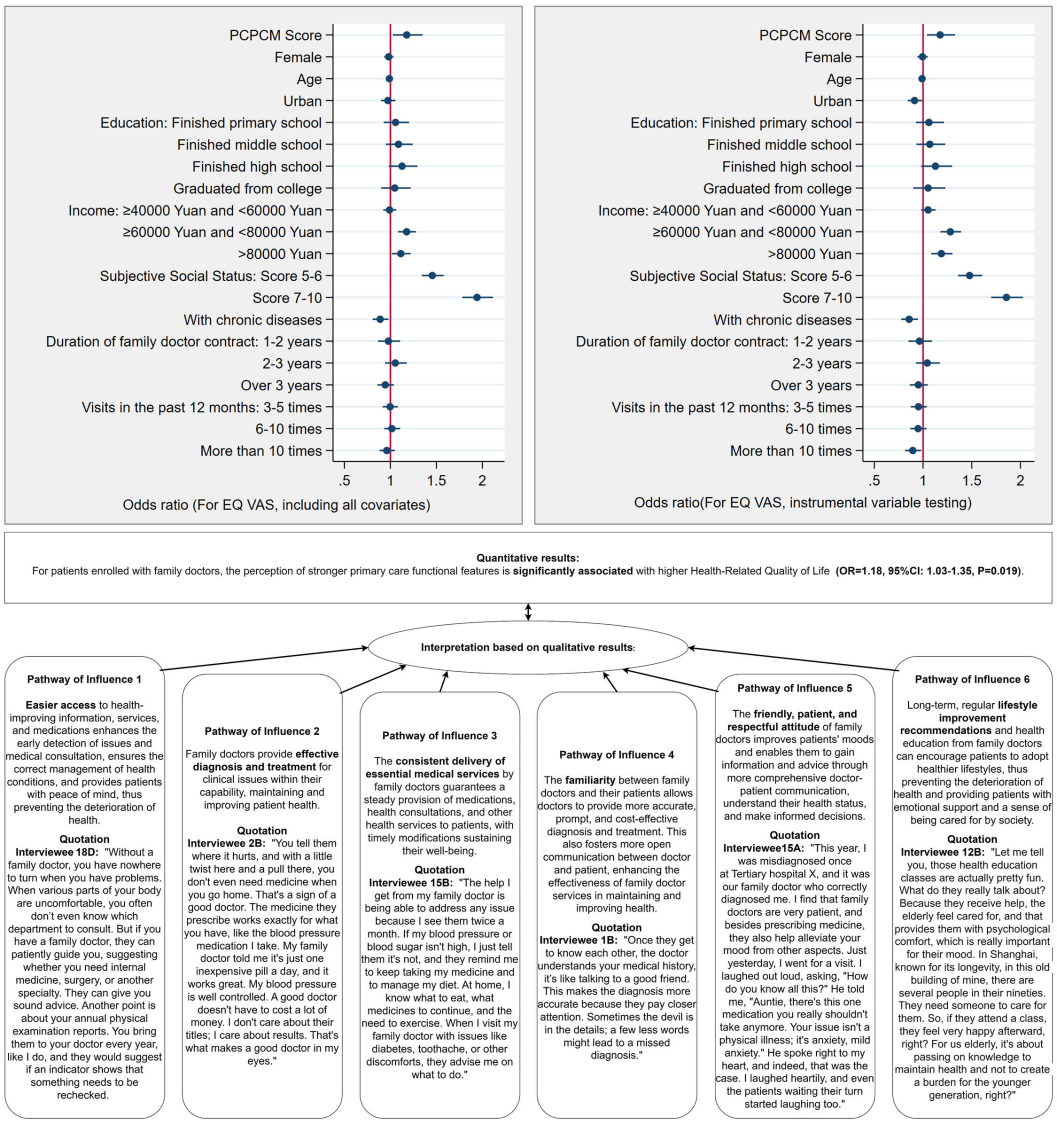
The relationship between primary care functional features and residents' health outcomes, and the pathways of influence.

**Figure 3:**
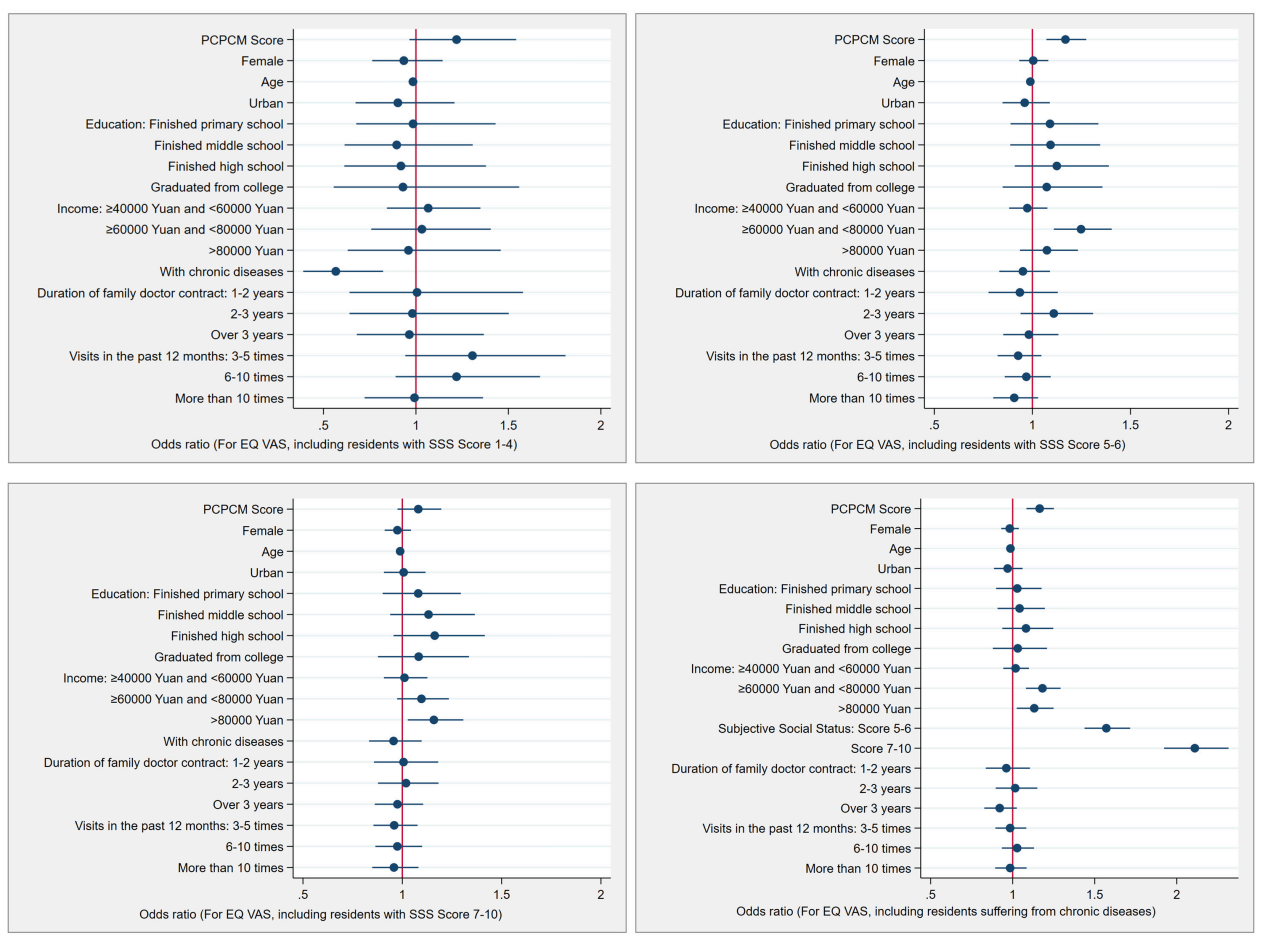
Subgroup analysis. The association between Person-Centered Primary Care Measure (PCPCM) Score and the EuroQol Visual Analogue Scale (EQ VAS) levels of patients with different subjective social status and chronic disease.

Regarding the relationship between primary care functional features and health (**Figure 2**), quantitative findings indicated that higher PCPCM scores were positively associated with increased EQ VAS levels (OR = 1.23; 95% CI = 1.14–1.32, *P* < 0.001). In regression models incorporating all necessary covariates, the OR slightly decreased (OR = 1.18; 95% CI = 1.03–1.35, *P* = 0.019). Under conditions meeting all instrumental variable tests, the 2SLS instrumental variable testing using the selected two instruments demonstrated a significant positive association between PCPCM scores and EQ VAS levels (OR = 1.18; 95% CI = 1.04–1.33, *P* = 0.009). In subgroup analyses, as shown in **Figure 3**, PCPCM scores also showed a significant positive association with EQ VAS levels in residents with middle SSS scores (OR = 1.17; 95% CI = 1.07–1.27, *P* < 0.001) and those with chronic diseases (OR = 1.17; 95% CI = 1.08–1.25, *P* < 0.001).

How local primary care functional features impact patient health outcomes is highlighted through six potential pathways presented in the qualitative results from patient narratives. These include improved access to information, services, and medications; effective diagnosis and treatment within a defined clinical scope; consistent provision of basic medical resources; established familiarity between patients and doctors; a friendly, patient, and respectful attitude; and the beneficial health effects of ongoing lifestyle modification guidance.

## DISCUSSION

The main findings of this mixed-methods study can be summarised in two key points. First, patients who utilised family doctors in Shanghai over the past year reported highly positive experiences regarding the functional features of primary care. These features are closely integrated with local practices and are shaped by the design of the local primary care system and the characteristics of the patient population. Second, higher levels of primary care functionality in local settings appear to be associated with better health outcomes among residents enrolled in family doctor services. The mechanisms behind these improved outcomes are multifaceted, involving clinical practices, health care services, the availability of medical resources, and doctor-patient relationships.

The PCPCM scores reported in this study exceeded those documented in surveys across 35 Organisation for Economic Cooperation and Development countries [56] and among primary care patients in Hong Kong [57]. While direct comparisons should be made with caution due to differing measurement tools, our scores also surpassed those from seven prior surveys using the PCAT conducted in various regions of China [24–31]. This suggests long-term progress in the development of primary care in Shanghai. Although publicly available data on the local government’s annual investment in primary care were not found, national statistics indicate that by 2022, Shanghai had 4.29 general practitioners per 10 000 residents, ranking second among China’s 31 provincial-level regions [58]. Moreover, a recent study revealed that 48.4% of Shanghai’s community health centres are equipped with CT scanners, and both drug availability and physician expertise are at high standards – far exceeding previous assessments of rural primary care in China [59,60]. In addition to these advancements, the Shanghai government has continuously introduced policies from 2013 to 2023 to enhance family doctor services and improve primary care functionality [61–64]. These policies include requiring family doctor teams to provide contracted services based on community units, with responsibilities divided by neighbourhood committees and allowing limited cross-district competition. The service scope of family doctors has been expanded to cover areas such as rehabilitation, long-term care, home visits, and palliative care, while allowing family doctors to prescribe medications based on specialist recommendations. Other initiatives include creating integrated electronic health records for contracted residents, offering online services such as virtual consultations and prescription deliveries, and reserving fast-track hospital appointments specifically for family doctor referrals.

The qualitative findings of this study demonstrate how local primary care services improve patients' experiences of primary care functions. Two notable examples highlight this. First, local family doctors often use WeChat (similar to WhatsApp) for direct communication with patients, offering timely health consultations, sometimes outside regular hours. This practice, while aligning with the Starfield Summit III framework [3] of access, expands the traditional notion of accessibility beyond appointment scheduling and standard care locations [5], enabling continuous contact between doctors and patients [21,65]. Second, ‘reverse coordination,’ mandated by local health regulations, requires specialists from tertiary hospitals to take part-time roles in primary care institutions [66]. Instead of referring patients upward, this approach brings specialists to the primary care level, improving the comprehensiveness of care and access to specialist services. Rooted in the dominant role of public health care institutions and government control, this practice supports primary care functionality. Additionally, many aspects highlighted in local health policies likely contributed to enhanced patient-reported functional experiences, such as timely appointment scheduling, stable referral channels, and the close relationships family doctors maintain with their communities and patient panels, as reflected in the qualitative findings.

It is important to note that, as a patient self-reported scale, the PCPCM results are closely tied to the respondents’ understanding of the ‘constructs’ being measured. As highlighted by some qualitative findings in this study that contradict the quantitative results, the high levels of primary care functionality reported by respondents are likely inflated, to some extent, due to the focus on actual users of family doctor services. This study primarily surveyed patients who had received family doctor services in the past year – a smaller subset than all contracted residents, those who have visited primary care facilities, or the broader community population theoretically covered by these services. This limitation can be attributed to the significant gap between the nominal coverage rates and actual utilisation of family doctor contracted services [22], which is partly driven by the government's strong administrative mandates and their interaction with the open health care market. As a result, tools like the PCPCM may have limited capacity to effectively measure the experiences of the entire contracted population in this context. This inflation may be particularly evident in domains such as comprehensiveness, integration, and doctor-patient relationships. Additionally, residents’ assessments of the PCPCM dimensions like ‘family orientation,’ ‘community orientation,’ and ‘goal orientation’ may be inflated due to cross-national differences in perceptions of health care services. For example, interventions addressing social determinants of health are not yet part of the government-mandated family doctor services [14,16,61–63]. With lower expectations in these areas, residents may be more impressed by family doctors' independent actions, leading to higher ratings. Considering the two issues mentioned above, we advise against directly comparing the functionality scores from this study with data from other countries without accounting for differences in health care systems and survey populations, especially when the samples in other countries' surveys may not fully represent the entire population of primary care patients [56].

Our qualitative research identified two new domains not covered by the 11 domains of the PCPCM: ‘being appreciated’ and ‘being cared for’. While the former was recognised at the Starfield Summit III [3], the latter has been largely overlooked in discussions on primary care functionality and has not been integrated into mainstream measurement outcomes and tools [3,6–9]. Our findings show that patients highly value respect and proactive care from medical staff with whom they feel familiar and close, addressing a critical emotional need – particularly for elderly patients who often face inadequate caregiving and social isolation [67]. These domains stand in stark contrast to the rushed and indifferent attitudes frequently encountered in large tertiary hospitals. This disparity is linked to China’s health care market, where government-controlled pricing keeps service costs similar across institutions, and large hospitals, leveraging their reputation, advanced equipment, and skilled doctors, continue to siphon off patients with common or chronic illnesses from primary care facilities [17]. This results in overburdened medical staff working in large hospitals, who struggle to provide personalised, attentive care, ultimately damaging patient experiences [68,69]. These findings suggest a potential strategy to rebalance primary and specialized care in China and similar health care systems. By encouraging family doctors – who have lower social status, smaller patient loads, and closer relationships with patients – to adopt more caring and proactive approaches, they can foster long-term trust and consultation habits with their contracted patients. Given the potential for these domains to influence patients' decisions to engage with primary care services in such environments, further cross-regional testing of these features is recommended. Based on broader and more robust evidence, the possibility of including them in primary care quality assessments in similar health care systems could also be considered.

Additionally, our research shows a correlation between higher PCPCM scores and better EQ VAS levels among residents, with similar results observed in subgroup analyses of chronic disease patients, aligning with several previous studies. These include the positive correlation between the accessibility of primary care services for chronic disease patients in China and their EQ VAS scores [46], as well as the association between higher PCPCM scores and better self-rated health in Massachusetts, USA [70]. However, the six pathways revealed by our qualitative findings exhibit both commonalities and differences when compared with one of the classic theories summarised by Starfield et al. [1]. Shared elements include accessibility, the quality of care for common diseases, the cumulative benefits of appropriate primary care over time, and the importance of early diagnosis and preventive advice. The key difference is that the mechanism for reducing unnecessary or inappropriate specialty care was not clearly reflected in our qualitative results. This may be due to the overall limited health literacy and medical knowledge among Chinese residents [71], as well as the absence of a gatekeeping system in primary care [17], which makes it difficult for patients to recognise the role of primary care in preventing inappropriate specialty care. Instead, as previously mentioned, one pathway highly valued by local residents is that some family doctors are perceived as ‘one of their own’ due to their friendly and approachable demeanour. This humanistic, egalitarian, and caring relationship fosters trust and encourages patients to openly communicate their health concerns. Previous studies have shown that open and thorough communication improves diagnostic accuracy and the effectiveness of medical care [72], and Chinese primary care patients have high expectations for receiving such services [73].

The implications of this study's findings can be summarised in two key areas. For policymakers and primary care service designers, the study demonstrates how a primary care system, built through strong political will but significantly eroded during economic hardship – similar to Cuba’s primary care system [74] – can be revitalised with increased government funding during times of economic growth, despite competition from specialised sectors in an open health care market. Shanghai’s response is clear: focus on chronic and common diseases by ensuring adequate health care resources through sound health policies, rebuilding the clinical capacity of general practitioners, and enhancing basic health care services with a focus on convenience, continuity, proactive care, and trust in doctor-patient relationships. Additionally, Shanghai should consider further enhancing the community and family orientation of its primary care services, an area where local functions still relatively fall short. For researchers, the findings suggest further exploring functional features shaped by China’s diverse primary care contexts across different provinces and rural areas. Evaluating the potential of these features to improve health care performance and health outcomes could lead to locally relevant quality metrics that help practitioners prioritise key aspects that may be overlooked in day-to-day practice.

The findings of this study come with several limitations. As one of China’s most economically developed regions [33] and an early pilot for primary care reforms since 2010 [34], Shanghai’s investment in local primary care, along with its human and equipment resources, is likely far greater than in regions like rural western provinces. Shanghai’s health policies regarding family doctor services are also more detailed and effectively implemented at the grassroots level compared to many other provinces [75-77]. Additionally, as a city with a rapidly aging population, Shanghai faces higher rates of chronic diseases and a greater demand for primary care services compared to younger cities like Shenzhen [56]. These differences suggest that the level of primary care functionality observed in this study is likely higher than in other regions of China, and the mechanisms influencing patient health may also vary. For instance, in remote areas lacking hospitals, the primary challenge is not competition with hospitals, but rather a shortage of medical personnel and resources, as seen in Zhejiang’s mountainous regions, where the government has addressed this issue by deploying mobile medical units that bring general practitioners to serve local residents [78]. Even within Shanghai, previous studies indicate that suburban and rural primary care patients may experience higher levels of functionality, likely due to increased government subsidies for non-central primary care institutions and less competition from large general hospitals [23]. Thus, while this study offers innovative insights for further exploration and research into primary care functionality and health outcomes in other regions of China, it should not be considered fully representative of the entire country, nor should it be assumed to have identified all functional features of primary care in China.

This study also faced several methodological limitations. The quantitative portion was limited by both the sample selection within the context of primary care and family doctor services in China, and the inherent constraints of the measurement tools, making it difficult to achieve full population representativeness. The multilevel model could benefit from further refinement of covariates to enhance the accuracy and robustness of the results. Additionally, the use of self-reported scales may have limitations in terms of objectivity and precision. Combined with sample selection constraints, this may have led to some overestimation of the primary care functionality levels measured in this study, and it also limits the robustness of the associations captured by the multilevel model. Furthermore, the cross-sectional survey could only identify associations, not establish causality. In the qualitative portion, while the ‘qualitative description’ method ensured data authenticity, it may have lacked depth. Furthermore, only 28.6% of respondents were willing to participate in interviews, and this low participation rate, combined with the limitations of the explanatory sequential design of the mixed-methods research, restricted the ability to capture the experiences of residents who signed up for family doctor services but did not use them. Building on the experiences outlined above, we plan to further explore primary care functionality in other regions of China, differentiating between various ‘primary care populations’ and designing targeted survey methods and content that reflect the diverse perceptions and experiences of different groups regarding primary care. This will help create a more comprehensive understanding of primary care functionality in China and inform its future quality assessment and improvement.

## CONCLUSIONS

This study reveals that family doctor-contracted services in Shanghai exhibit high functional feature scores. However, the practices and services that generate these features are deeply shaped by the local social and health care contexts. Additionally, higher functionality may be associated with improved population health outcomes. Beyond improved access to information, services, and medications; effective diagnosis and treatment within a designated clinical scope; and consistent provision of basic medical resources, these effects may also stem from established familiarity between patients and doctors, friendly,patient, and respectful attitude, and the beneficial health effects of ongoing lifestyle modification guidance. This underscores the necessity of further exploring primary care functional features across broader regions of China and necessitates the development of tools that are more tailored to local conditions to more accurately assess primary care functional features and support quality improvement.

## Additional material


Online Supplementary Document
