# Supplementary material for: Primary care functional features and their health impact on patients enrolled in the Shanghai family doctor service: a mixed-methods study
Source: J Glob Health. 2025 Jan 10;15:04007. doi: 10.7189/jogh.15.04007 (PMC11719843; doi:10.7189/jogh.15.04007)
Supplement: Online Supplementary Document [file jogh-15-04007-s001.pdf]

ONLINE SUPPLEMENTARY DOCUMENT

Title: Primary Care Functional Features and Their Health Impact on Patients Enrolled in the Shanghai Family Doctor Service: A Mixed-Methods Study

Authors: Yang Wang, Hua Jin, Hui Yang, Yang Zhao, Yi Qian, Dehua Yu, Hai Fang

*** Correspondence to:**

Prof. Dehua Yu

Department of General Practice, Research Center for General Practice, Yangpu Hospital, School of Medicine, Tongji University, Shanghai, China

Email: shgprc@yeah.net

Tel.: +86 02165690520

Prof. Hai Fang

China Center for Health Development Studies, Peking University, Beijing, China

Email: hfang@hsc.pku.edu.cn

Tel.: +86 10 82805702

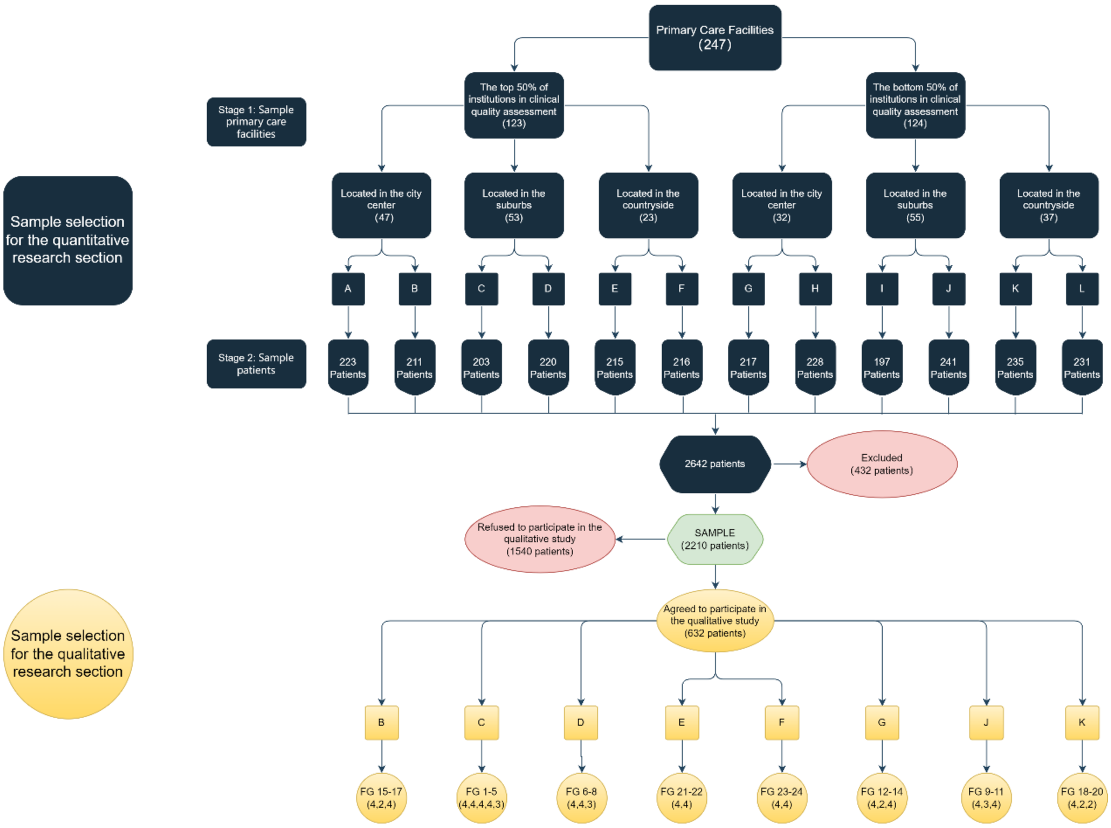

Figure S1. The sampling strategy for mixed method research

Note: FG: Focus groups.

Table S1 Results of psychometric analyses of PCPCM

**Internal consistency reliability(Cronbach's α)**	0.94
**Stability reliability (ICC)**	0.56
**Homogeneity (Spearman's Rank-Order Correlation)**	
**Item1**	0.69*
**Item2**	0.71*
**Item3**	0.77*
**Item4**	0.68*
**Item5**	0.79*
**Item6**	0.67*
**Item7**	0.85*
**Item8**	0.84*
**Item9**	0.78*
**Item10**	0.78*
**Item11**	0.78*
**Construct-related validity (Spearman's Rank-Order Correlation)**	0.72*
**Criterion-related validity (Spearman's Rank-Order Correlation)**	0.54*
**Dimensionality**	
**Proportion of Variance Explained by Principal Component 1**	0.65
**Proportion of Variance Explained by other Components**	0.01-0.08
**Eigenvalue of Principal Component 1**	7.17
**Eigenvalues of Other Components**	0.16-0.90
**Model fit (Rasch Analysis)**	0.01
**Infit Mean Square (Person)**	0.99
**Outfit Mean Square (Person)**	1.04
**Infit Mean Square (Item)**	0.96
**Outfit Mean Square (Item)**	1.04

Note: *:P＜0.001; ICC: Intraclass Correlation Coefficient

Table S2. Details of the covariates

Variable	Categorical/numerical range	Measurement method
**Covariate**		
**Gender**	Male<br>Female	Investigator observation
**Age**	< 35 years<br>≥35 years and <55 years<br>≥55 years and <65 years<br>≥65 years and <75 years<br>≥75 years	Electronic health record
**Household registration (Hukou)**	Rural<br>Urban	Patient's response to the questions
**Education**	Didn't finish primary school<br>Finished primary school<br>Finished middle school<br>Finished high school<br>Graduated from college	Patient's response to the questions
**Income**	<40000 Yuan<br>≥40000 Yuan and <60000 Yuan<br>≥60000 Yuan and <80000 Yuan<br>>80000 Yuan	Patient's response to the questions
**Subjective Social Status**	Score 1-4<br>Score 5-6<br>Score 7-10	Patient's response to the questions (MacArthur Scale of Subjective Social Status)
**Suffered chronic diseases**	Without chronic diseases<br>With chronic diseases	Electronic health record (Records on hypertension, diabetes, dyslipidemia, coronary heart disease, angina, myocardial infarction, stroke, stomach or digestive system diseases, cancer, chronic obstructive pulmonary disease, chronic bronchitis, emphysema, cor pulmonale, liver diseases, kidney diseases, arthralgia, rheumatism, or asthma)
**Physical activity**	Does not meet WHO recommended levels<br>Meets WHO recommended levels	Patient's response to the questions
**Alcohol consumption**	Do not drink alcohol<br>Drink less than one drink per day<br>Drink 1 or more drinks per day	Patient's response to the questions
**Smoking status**	No smoking<br>Low dependence<br>Moderate or high dependence	Patient's response to the questions (The Fagerström Test for Nicotine Dependence)
**Obesity status**	Not overweight<br>Overweight or obese	Electronic health record (BMI)
**Health insurance**	No medical insurance<br>Moderate-coverage insurance scheme (This category includes urban and rural resident medical insurance only or urban resident medical insurance only)<br>High-coverage insurance scheme (This category includes urban employee medical insurance only, government medical insurance only, private medical insurance purchased by the work unit only, or private medical insurance purchased by the individual only)	Patient's response to the questions
**Visits in the past 12 months**	Within 1 year<br>1-2 years<br>2-3 years<br>Over 3 years	Electronic health record
**Duration of family doctor contract**	1-2 times<br>3-5 times<br>6-10 times<br>More than 10 times	Electronic health record

Table S3. Details of the instrumental variables

Instrumental variables	The rationale for selecting it as an instrumental variable.	The results of the Spearman regression with the key variables	The results of the instrumental variable validity tests.
**Does the family doctor provide services during the weekend**	Whether family doctors provide medical services on weekends does not have a clear direct relationship with patient health in a healthcare system where patients can freely choose their providers, especially since most general hospitals in China are open on weekends. However, it improves patients perceived functional features, particularly **accessibility**, thereby enhancing health outcomes through more accessible primary care services (see Pathway 1 in Figure 2).	**With the independent variable:**<br>Spearman's rho = 0.5046<br>$P \leqslant 0.001$<br><br>**With the outcome variable:**<br>Spearman's rho = 0.0261<br>P= 0.1801	**Endogeneity Test:**<br>Durbin $\chi^2(1) = 9.12$<br>p = 0.0025<br><br>Wu-Hausman F(1,2380) = 9.14<br>p = 0.0025<br><br>**Weak Instrumental Variables Test:**<br>F(2,2380) = 322.84<br>Minimum eigenvalue statistic = 322.84<br><br>**Overidentification Test:**<br>Sargan $\chi^2(1) = 0.41$<br>p = 0.5233<br><br>Basmann $\chi^2(1) = 0.41$<br>p = 0.5235
**Does the family doctor offer telephone health consultations to patients after hours**	Similarly, whether family doctors offer telephone consultations does not have a clear direct relationship with patient health, but it also enhances perceived functional features, particularly **accessibility**, leading to improved health outcomes through more accessible primary care services (see Pathway 1 in Figure 2).	**With the independent variable:**<br>Spearman's rho = 0.4706<br>$P \leqslant 0.001$<br><br>**With the outcome variable:**<br>Spearman's rho =-0.0043<br>P= 0.8265	

Note: Accessibility is theoretically considered the most fundamental attribute of primary care functional features. Without accessibility, other functional features cannot exist; therefore, it is often **closely associated with** the overall level of primary care functional features (Reference: Mold JW. From the North American Primary Care Research Group: how primary care produces better outcomes–a logic model. Ann Fam Med. 2014;12(5):483).

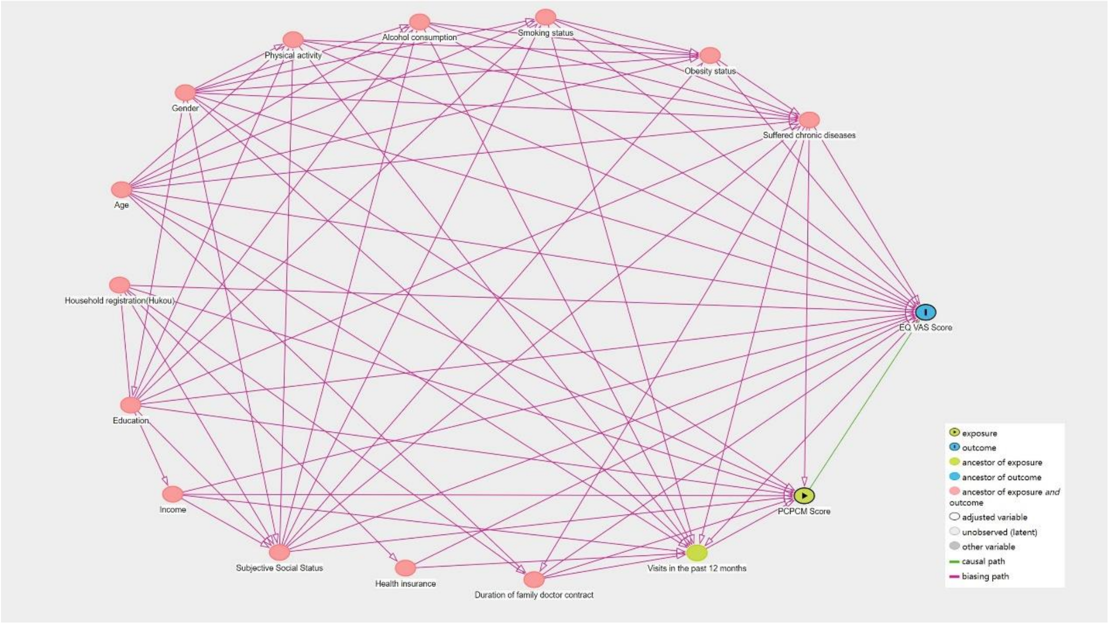

Figure S2. Causal directed acyclic graphs

Note: Minimal sufficient adjustment sets for estimating the total effect of PCPCM Score on EQ VAS: Gender, Age, Household registration (Hukou), Education, Income, Subjective Social Status, Suffered chronic diseases, Duration of family doctor contract, Visits in the past 12 months.

Table S4. Characteristics of residents participating in focus group interviews

Interviewee ID	Gender	Age	EQ VAS Score	Suffered chronic diseases	Education	Subjective Social Status	Number of visits to the family doctor in the past 12 months	Duration of contract with the family doctor	PCPCM Score
**1A**	Male	71	94	With chronic diseases	Finished middle school	Score 7-10	6-10 times	Over 3 years	3.91
**1B**	Female	69	91	Without chronic diseases	Finished middle school	Score 7-10	6-10 times	Over 3 years	4.00
**1C**	Male	75	92	With chronic diseases	Finished middle school	Score 7-10	6-10 times	Over 3 years	4.00
**1D**	Female	72	91	With chronic diseases	Finished middle school	Score 7-10	6-10 times	Over 3 years	4.00
**2A**	Male	33	92	Without chronic diseases	Graduated from college	Score 7-10	1-2 times	Over 3 years	3.64
**2B**	Female	56	92	With chronic diseases	Finished high school	Score 7-10	3-5 times	Over 3 years	3.73
**2C**	Male	79	90	With chronic diseases	Finished middle school	Score 7-10	6-10 times	Over 3 years	3.45
**2D**	Female	77	92	With chronic diseases	Finished middle school	Score 7-10	More than 10 times	Over 3 years	3.45
**3A**	Female	59	77	With chronic diseases	Finished middle school	Score 5-6	More than 10 times	Over 3 years	2.36
**3B**	Male	78	71	With chronic diseases	Finished middle school	Score 5-6	More than 10 times	Over 3 years	4.00
**3C**	Female	74	60	With chronic diseases	Finished middle school	Score 7-10	More than 10 times	Over 3 years	4.00
**3D**	Female	70	60	With chronic diseases	Finished primary school	Score 5-6	More than 10 times	Over 3 years	3.91
**4A**	Female	73	83	With chronic diseases	Graduated from college	Score 7-10	1-2 times	Over 3 years	3.00
**4B**	Male	71	80	With chronic diseases	Graduated from college	Score 7-10	6-10 times	Over 3 years	3.55
**4C**	Male	63	61	With chronic diseases	Finished high school	Score 7-10	6-10 times	Over 3 years	4.00
**4D**	Female	66	70	With chronic diseases	Graduated from college	Score 5-6	3-5 times	Over 3 years	4.00
**5A**	Female	61	63	With chronic diseases	Finished high school	Score 7-10	More than 10 times	Over 3 years	2.73
**5B**	Female	72	81	With chronic diseases	Finished high school	Score 7-10	More than 10 times	2-3 years	4.00
**5C**	Female	72	64	With chronic diseases	Finished high school	Score 7-10	3-5 times	Over 3 years	2.91
**6A**	Female	49	70	With chronic diseases	Graduated from college	Score 7-10	More than 10 times	Over 3 years	4.00
**6B**	Male	81	90	With chronic diseases	Finished high school	Score 5-6	More than 10 times	Over 3 years	4.00
**6C**	Female	73	67	With chronic diseases	Finished middle school	Score 7-10	1-2 times	Over 3 years	4.00
**6D**	Female	65	40	With chronic diseases	Finished high school	Score 1-4	More than 10 times	Over 3 years	3.91

**7A**	Female	58	82	With chronic diseases	Finished high school	Score 7-10	More than 10 times	2-3 years	3.91
**7A**	Male	63	77	With chronic diseases	Finished high school	Score 7-10	More than 10 times	1-2 years	4.00
**7B**	Male	69	59	With chronic diseases	Finished high school	Score 1-4	More than 10 times	Over 3 years	3.45
**7C**	Male	68	80	With chronic diseases	Graduated from college	Score 7-10	6-10 times	2-3 years	3.91
**8A**	Female	73	49	With chronic diseases	Finished middle school	Score 5-6	More than 10 times	Over 3 years	3.55
**8B**	Male	75	0	With chronic diseases	Finished high school	Score 5-6	More than 10 times	2-3 years	3.27
**8C**	Female	71	80	With chronic diseases	Finished middle school	Score 7-10	More than 10 times	Over 3 years	4.00
**9A**	Female	71	70	With chronic diseases	Finished primary school	Score 5-6	3-5 times	Over 3 years	4.00
**9B**	Male	60	78	With chronic diseases	Finished middle school	Score 5-6	1-2 times	Over 3 years	3.55
**9C**	Female	69	80	With chronic diseases	Finished primary school	Score 1-4	3-5 times	Over 3 years	4.00
**9D**	Female	71	80	With chronic diseases	Finished high school	Score 5-6	6-10 times	Over 3 years	3.73
**10A**	Male	75	70	With chronic diseases	Finished high school	Score 5-6	More than 10 times	Over 3 years	4.00
**10B**	Male	76	80	With chronic diseases	Finished high school	Score 1-4	6-10 times	Over 3 years	3.18
**10C**	Male	74	100	With chronic diseases	Finished high school	Score 5-6	3-5 times	Over 3 years	3.18
**11A**	Female	63	59	With chronic diseases	Finished high school	Score 1-4	3-5 times	Over 3 years	2.09
**11B**	Male	80	40	With chronic diseases	Finished primary school	Score 1-4	1-2 times	Over 3 years	2.54
**11C**	Female	76	75	With chronic diseases	Finished primary school	Score 7-10	3-5 times	Over 3 years	3.73
**11D**	Female	75	80	With chronic diseases	Finished middle school	Score 7-10	More than 10 times	Over 3 years	4.00
**12A**	Male	73	81	With chronic diseases	Finished high school	Score 5-6	More than 10 times	Over 3 years	3.82
**12B**	Female	39	90	Without chronic diseases	Graduated from college	Score 5-6	1-2 times	2-3 years	4.00
**12C**	Female	48	49	With chronic diseases	Graduated from college	Score 5-6	1-2 times	Over 3 years	3.82
**12C**	Male	21	42	Without chronic diseases	Graduated from college	Score 5-6	1-2 times	Over 3 years	3.55
**12D**	Female	58	100	With chronic diseases	Graduated from college	Score 5-6	1-2 times	2-3 years	3.91
**13A**	Male	58	82	With chronic diseases	Finished middle school	Score 5-6	More than 10 times	2-3 years	3.73
**13B**	Male	66	42	With chronic diseases	Finished high school	Score 5-6	1-2 times	Within 1 year	3.00
**14A**	Female	71	75	With chronic diseases	Finished middle school	Score 7-10	6-10 times	Over 3 years	3.73

**14B**	Female	68	47	With chronic diseases	Finished high school	Score 5-6	More than 10 times	Over 3 years	3.64
**14C**	Female	70	81	With chronic diseases	Finished middle school	Score 5-6	More than 10 times	2-3 years	3.82
**14D**	Female	60	36	With chronic diseases	Finished middle school	Score 5-6	More than 10 times	Over 3 years	4.00
**15A**	Female	71	80	With chronic diseases	Graduated from college	Score 7-10	More than 10 times	Over 3 years	4.00
**15B**	Male	66	80	With chronic diseases	Finished high school	Score 5-6	More than 10 times	Within 1 year	3.00
**15C**	Female	60	85	With chronic diseases	Finished high school	Score 5-6	More than 10 times	Over 3 years	4.00
**15D**	Female	71	70	With chronic diseases	Finished primary school	Score 7-10	6-10 times	Over 3 years	3.18
**16A**	Male	60	91	With chronic diseases	Finished primary school	Score 5-6	6-10 times	Over 3 years	4.00
**16B**	Male	61	80	With chronic diseases	Finished high school	Score 5-6	More than 10 times	Within 1 year	4.00
**17A**	Male	76	80	With chronic diseases	Graduated from college	Score 5-6	More than 10 times	Over 3 years	4.00
**17B**	Female	75	70	With chronic diseases	Finished middle school	Score 5-6	More than 10 times	Over 3 years	4.00
**17C**	Male	74	82	With chronic diseases	Finished primary school	Score 7-10	6-10 times	Over 3 years	4.00
**17D**	Female	55	96	With chronic diseases	Graduated from college	Score 5-6	1-2 times	2-3 years	4.00
**18A**	Female	53	66	With chronic diseases	Finished middle school	Score 5-6	1-2 times	Over 3 years	3.09
**18B**	Female	65	73	With chronic diseases	Finished middle school	Score 5-6	6-10 times	Over 3 years	2.55
**18C**	Female	68	29	With chronic diseases	Finished middle school	Score 1-4	6-10 times	Over 3 years	2.82
**18D**	Female	52	72	With chronic diseases	Graduated from college	Score 7-10	6-10 times	2-3 years	3.36
**19A**	Male	53	61	With chronic diseases	Finished high school	Score 5-6	6-10 times	Over 3 years	3.00
**20A**	Female	51	47	With chronic diseases	Finished middle school	Score 5-6	6-10 times	Over 3 years	3.18
**20B**	Female	52	25	With chronic diseases	Finished middle school	Score 1-4	6-10 times	Over 3 years	2.91
**21A**	Male	69	83	With chronic diseases	Finished primary school	Score 7-10	6-10 times	Over 3 years	3.91
**21B**	Female	64	81	Without chronic diseases	Finished high school	Score 1-4	1-2 times	2-3 years	4.00
**21C**	Female	69	81	With chronic diseases	Finished middle school	Score 5-6	6-10 times	1-2 years	4.00
**21D**	Male	68	0	With chronic diseases	Finished middle school	Score 1-4	More than 10 times	Over 3 years	4.00
**22A**	Female	73	70	With chronic diseases	Didn't finish primary school	Score 1-4	3-5 times	2-3 years	2.18
**22B**	Female	60	39	With chronic diseases	Finished high school	Score 5-6	3-5 times	Over 3 years	4.00

**22C**	Female	68	100	With chronic diseases	Finished primary school	Score 5-6	1-2 times	2-3 years	4.00
**22D**	Female	75	97	With chronic diseases	Finished high school	Score 7-10	6-10 times	Over 3 years	4.00
**23A**	Male	69	72	With chronic diseases	Finished primary school	Score 7-10	1-2 times	1-2 years	3.00
**23B**	Female	66	71	With chronic diseases	Finished middle school	Score 1-4	3-5 times	Over 3 years	4.00
**23C**	Female	69	80	With chronic diseases	Finished primary school	Score 7-10	3-5 times	Within 1 year	3.00
**23D**	Female	61	80	With chronic diseases	Finished primary school	Score 1-4	6-10 times	Over 3 years	3.91
**24A**	Female	38	78	With chronic diseases	Graduated from college	Score 1-4	1-2 times	Over 3 years	4.00
**24B**	Female	78	79	With chronic diseases	Finished primary school	Score 1-4	6-10 times	Over 3 years	4.00
**24C**	Female	37	85	With chronic diseases	Graduated from college	Score 5-6	1-2 times	Within 1 year	3.91
**24D**	Female	35	75	With chronic diseases	Graduated from college	Score 5-6	1-2 times	Over 3 years	4.00

Table S5. Topics discussed in focus group interviews with residents signed up with family doctors

Stage of the question	Main topics of discussion
**Opening and introductory questions**	The main threats and issues facing patients' health.<br><br>The most prominent feeling when seeking healthcare from a family doctor in the past year.
**Key questions**	Who people prioritize contacting for health help and why.<br><br>Ease and experience of accessing family doctors.<br><br>Accuracy and capabilities of family doctors in diagnosing and treating health issues.<br><br>Comprehension and management of patients' health by family doctors.<br><br>Impact of long-term relationship and familiarity with family doctors.<br><br>Comparison of seeing family doctors vs. Specialists first.<br><br>Family doctors' efforts in referring patients to specialist clinics at large hospitals.<br><br>Patients' trust and satisfaction with family doctors.<br><br>Family doctors' role in community health and public health education.<br><br>Cost and financial implications of consulting family doctors vs. Specialists.
**Ending questions**	The main aspects in which family doctors are most helpful in maintaining and improving their health, in the eyes of the patients.<br><br>The potential impact on patients' health conditions if they lose their family doctor.
